# Organic manures and inorganic fertilizers effects on soil properties and economic analysis under cassava cultivation in the southern Cameroon

**DOI:** 10.1038/s41598-022-17991-6

**Published:** 2022-11-29

**Authors:** Eloi Gervais Bilong, Monique Abossolo-Angue, Lawrence Tatanah Nanganoa, Bienvenu Désiré Anaba, Francis Ngome Ajebesone, Birang À. Madong, Paul Bilong

**Affiliations:** 1 Institute of Agricultural Research for Development (IRAD), Messa, P.O. Box 2123, Yaoundé, Cameroon; 2 Department of Earth Sciences, Faculty of Science, University of Yaounde 1, P. O. Box 812, Yaoundé, Cameroon

**Keywords:** Plant sciences, Environmental sciences

## Abstract

Cassava cultivation causes serious soil fertility depletion in southern Cameroon due to high mining of soil nutrients by the crop. This study aimed to evaluate the effects of *Tithonia diversifolia* fresh biomass (TB), poultry manure (PM) and inorganic fertilizers (IF) on soil properties, cassava yield, and the economic returns. The treatments consisted of two rates of TB (10 and 20 t ha^−1^), two rates of PM (10 and 20 t ha^−1^), two rates of combined TB and PM (5 and 10 t ha^−1^), a single rate of inorganic fertilizers (100 N:22P:83 K kg ha^−1^) and a control. The results showed that soil properties, soil quality index and cassava yield were significantly improved by the application of the organic manures. *Tithonia diversifolia* fresh biomass (TB) and poultry manure (PM) lowered the soil bulk density, increased soil total porosity, water holding capacity and chemical properties. TB and PM, solely or mixed, improved the aerial dry biomass (ADB) and fresh tuber yield (FTY) of cassava. The organic manures performed better than inorganic fertilizer. The highest yield (51 and 52 t ha^−1^ of fresh tubers) was obtained with the mixture of TB and PM applied at 10 t ha^−1^ each for the successive years. Positive and significant correlation was found between SQI and cassava yield. TB and PM combined at 10 t ha^−1^ each was the most profitable and cost-effective treatment, with a good benefit:cost ratio of 3.2:1 and net return of FCFA 3.736.900 ha^−1^. Thus, the use of *Tithonia diversifolia* fresh biomass and poultry manure is a sustainable method for cassava production in the southern Cameroon.

## Introduction

Cassava (*Manihot esculenta* Crantz) is one of the strategic crops of the African continent, given its significant contribution to farmers' livelihoods and its potential to transform African economies^1,2^. As a root crop grown in the tropics by over 800 million people, cassava can grow with minimal inputs in marginal soil conditions and in drought-prone areas^3,4^.

Cassava can adapt to diverse climatic conditions, survive long dry spells, and can be harvested and stored on a flexible time schedule, all of which qualifies cassava as a food security crop in sub-Saharan Africa^5,6^. The crop has great potential to contribute to African development and is increasing its income-earning potential for small-scale farmers and related value chains on the continent^7^. Another importance of cassava derives from the fact that it has become an industrial crop, which is processed into different products, including bread, pasta, and couscous-like products^1^. In addition to the food industry, cassava starch is used for textiles, paper industry, manufacture of plywood, veneer adhesives, glucose and dextrin syrups^1^.

In Cameroon, cassava is grown in all of the five agro-ecological zones^8^. Approximately 80% of its production comes from the humid forest zone^9^. In many parts of the country, the leaves and tender shoots are also eaten as vegetables^10^. The Cameroon economy remains heavily dependent on the agricultural sector, which employs more than 68% of the national workforce, and provides about 15% of the public budget^11^. Cassava is grown by 75% of smallholder farmers in the forest zone^12,13^. Yields are very low and generally below 17 tons ha^-1^.

Despite the fact that cassava grows well even on poor soils, its continuous cultivation in the same area without minimal inputs and good practices can result in significant deterioration of soil productivity due to the high removal of soil nutrients by the crop^14^. The application of inorganic fertilizers could increase cassava production on smallholder farms. However, the high cost of inorganic fertilizers coupled with the limited resources of farmers in the forest zone of Cameroon make their adoption difficult. Ever since, organic manures have been used as alternative to inorganic fertilizers because of their beneficial effects on soil productivity^8,15^. These effects include improvement of soil physical and chemical parameters^16,17^.

However, few studies on soil fertility management on cassava farms have been undertaken in Cameroon on cassava yield after applying organic manures and inorganic fertilizers^18^ but little is known on their effects on soil physical and chemical properties, and soil quality index. Therefore, the objective of this study was to explore the effects of *Tithonia diversifolia* fresh biomass (TB), poultry manure (PM) and inorganic fertilizer (IF) application on soil physical and chemical properties, soil quality index and yield of cassava in the humid forest zone of southern Cameroon. The economic returns of these farming systems were also studied.

## Results

### Manures quality and initial soil properties

The chemical analysis of *Tithonia diversifolia* fresh biomass and poultry manure showed that poultry manure had higher pH compared to *Tithonia diversifolia* fresh biomass (Table 1). Among the two organic manures, *Tithonia diversifolia* fresh biomass had the highest content in N, K and Ca. whereas poultry manure had the highest C and P concentrations and C/N ratio. The initial soil physical parameters of the study site and the chemical properties of the organic manures used (TB and PM) are shown in Table 2. The soil profile described in the site belongs to ferralsols group according to WRB^19^, with sandy clay texture. Its moderate bulk density (1.2 g·cm^−3^), total porosity (54.7%) and water holding capacity (37.5%) revealed its suitability to roots penetration and cassava cultivation.

**Table 1:** Chemical properties of *T. diversifolia *fresh biomass and poultry manure.

Materials	OC (%)	N (%)	C/N ratio	P (%)	K (%)	Ca (%)	Mg (%)
*Thitonia* fresh biomass	24.8^b^	3.47^a^	7.15^b^	0.6^b^	3.8^a^	3.06^a^	0.54^a^
Poultry manure	36.2^a^	2.53^b^	14.3^a^	1.3^a^	2.1^b^	1.52^b^	0.6^a^

Values followed by similar letters under the same column are not significantly different at p = 0.05 according to Duncan’s multiple range test.

**Table 2:** Initial soil characteristics of the study sites.

Soil characteristics	Essong-Mintsang
Texture class	Sandy clay
Bulk density (g cm^−3^)	1.2
Water holding capacity (%)	37.5
pH (water)	5.6
Organic matter (%)	2.6
Total N (%)	0.14
C:N ratio	10.8
Available P (mg kg^−1^)	4.1
Exchangeable K (cmol kg^−1^)	0.16
Exchangeable Ca (cmol kg^−1^)	0.49
Exchangeable Mg (cmol kg^−1^)	0.37
CEC (cmol kg^−1^)	5.6

### *Tithonia diversifolia* fresh biomass and poultry manure effect on soil physical properties

The soil physical properties (bulk density, total porosity and water holding capacity) for the different treatments applied are shown in Table 3. Organic amendements had a significant influence on these soil physical properties.

**Table 3:** Effect of treatments on soil physical properties in 2016/2017 and 2017/2018 cropping seasons.

Treatments code	BD (g·cm^−3^)		TP (%)		WHC (%)	
	2016/2017	2017/2018	2016/2017	2017/2018	2016/2017	2017/2018
T0	5.4^d^	5.0^d^	0.15^e^	0.1^e^	0.27^e^	0.18^e^
T1	5.3^d^	4.9^d^	0.28^d^	0.2^d^	0.22^e^	0.19^e^
T2	7.4^b^	7.9^b^	0.53^a^	0.57^ab^	0.6^bc^	0.75^ab^
T3	6.7^c^	7.1^c^	0.36^c^	0.38^c^	0.44^d^	0.55^d^
T4	8^a^	8.5^a^	0.55^a^	0.53^b^	0.61^b^	0.69^bc^
T5	6.9^c^	7.3^c^	0.39^bc^	0.38^c^	0.55^bc^	0.62^cd^
T6	8^a^	8.5^a^	0.58^a^	0.6^a^	0.75^a^	0.83^a^
T7	7.2^b^	7.7^b^	0.41^b^	0.4^c^	0.51^cd^	0.57^d^
Sig	*		*		*	

Values followed by similar letters under the same column are not significantly different at p = 0.05. T0 = control (no amendement), T1 = IF: 13-13-23 NPK at 450 t ha^−1^ + urea at 100 t ha^−1^, T2 = TB at 20 t ha^−1^, T3 = TB at 10 t ha^−1^, T4 = PM at 20 t ha^−1^, T5 = PM at 10 t ha^−1^, T6 = TB at 10 t ha^−1^ + PM at 10 t ha^−1^, T7 = TB at 5 t ha^−1^ + PM at 5 t ha^−1^.

*TM* *Tithonia diversifolia* fresh biomass, *PM*  poultry manure, *IF*  inorganic fertilizer.

*Significant difference at p = 0.05 ; *ns* not significant at 0.05.

Soil bulk density decreased and total porosity and water holding capacity improved significantly when *Tithonia diversifolia* fresh biomass (TB) and poultry manure (PM), were applied either solely or combined compared to the control (T0) and inorganic fertilizer (T1) treatments. Increasing the rate of organic manures increased soil water holding capacity and total porosity and decreased soil bulk density. The lowest bulk density, the highest total porosity and the highest water holding capacity was recorded with PM applied at 20 t ha^−1^ (T4) and TB + PM applied at 10 t ha^−1^ each (T6). Over the cropping seasons, sole TB and PM, and their combination at different rates reduced soil bulk density in the range from 14 to 26%, increased total porosity in the range from 10 to 16% and water holding capacity from 13 to 30% as compared to the control (T0). Inorganic fertilizer (T1) had no significant effect on soil physical parameters as compared to the control treatment. Soil physical properties were better improved in the 2017/2018 cropping season compared to the 2016/2017 cropping season. However, taken as individual factors, cropping season (S) was not significant for bulk density (BD), total porosity (TP) and water holding capacity (WHC). Treatment (T) was significant for all studied soil physical parameters (BD, TP and WHC). The S × T interaction was not significant for soil bulk density, total porosity and water holding capacity.

### *Tithonia diversifolia* fresh biomass and poultry manure effect on soil chemical properties

**Figure 1:**
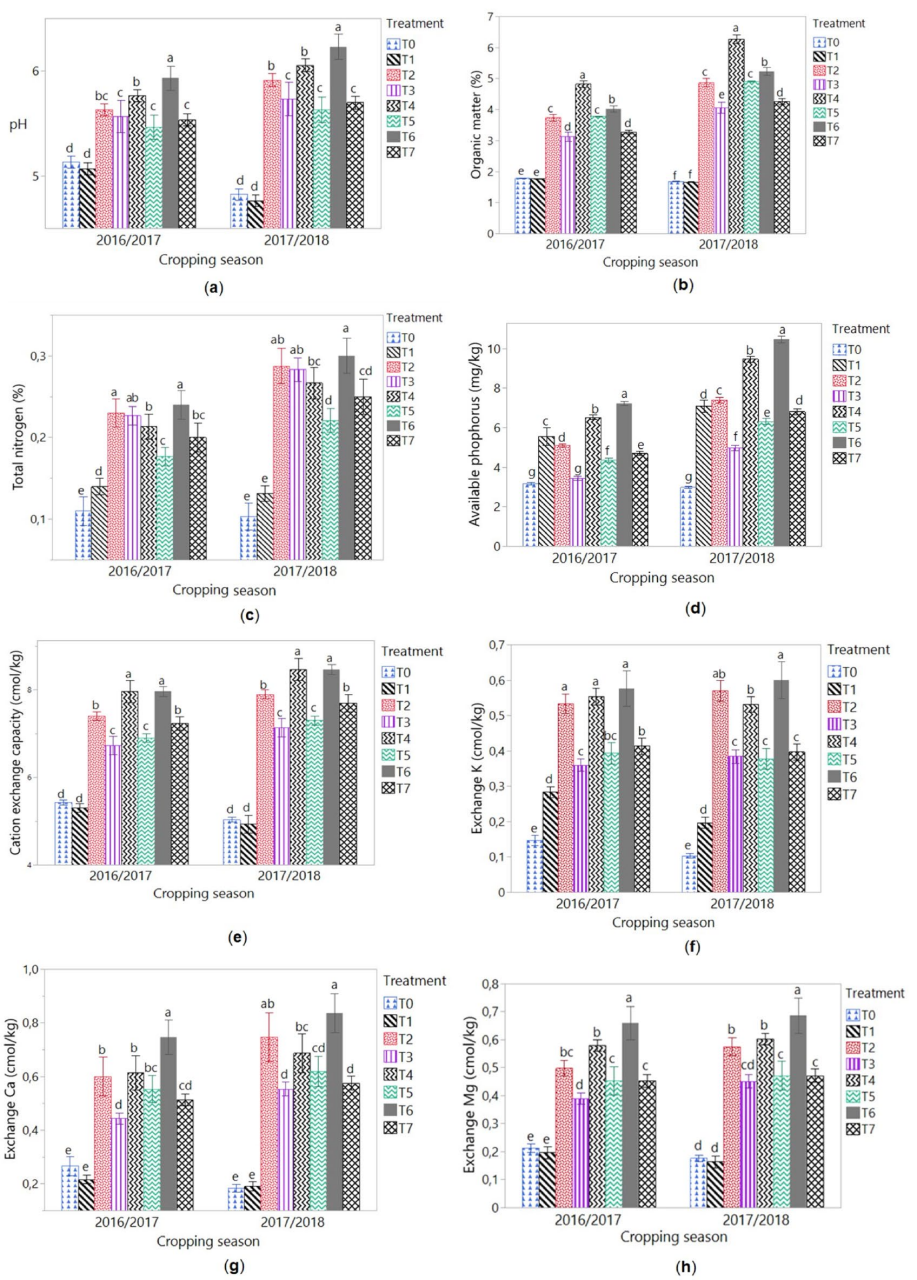
Means of soil chemical properties in 2016/2017 and 2017/2018 cropping seasons. T0 = control (no amendement), T1 = IF : 13-13-23 NPK at 450 t ha^−1^ + urea at 100 t ha^−1^, T2 = TB at 20 t ha^−1^, T3 = TB at 10 t ha^−1^, T4 = PM at 20 t ha^−1^, T5 = PM at 10 t ha^−1^, T6 = TB at 10 t ha^−1^ + PM at 10 t ha^−1^, T7 = TB at 5 t ha^−1^ + PM at 5 t ha^−1^. *TM* *Tithonia diversifolia* fresh biomass, *PM* poultry manure, *IF* inorganic fertilizer. Values followed by similar letters are not significantly different at p = 0.05.

Treatments with *Tithonia diversifolia* green manure (TB) and poultry manure (PM) affected soil chemical properties at both experimental seasons (Fig. 1). There were significant differences in soil chemical properties between the cropping seasons, likewise significant differences on chemical properties were observed between all the treatments. Application of TB and PM solely or mixed (T2, T3, T4, T5, T6, and T7) increased soil pH, OM, total N, available P, CEC and exchangeable cations (K, Ca and Mg) as compared to the control (T0). while the treatment with inorganic fertilizer (T1) decreased the soil pH and OM, and increased total N and available P (Table 4). No significant differences were noted in soil CEC between the inorganic fertilizers and control treatments. However, increase in exchangeable K were noted with the application of inorganic fertilizers. For the overall cropping seasons, the application of TB and PM solely or mixed increased the soil pH, OM, total N, available P, CEC, exchangeable K, exchangeable Ca and exchangeable Mg in the range from 13 to 23%, 108 to 188%, 92 to 159%, 68 to 188%, 33 to 89%, 210 to 394%, 140 to 270% and 79 to 249%, respectively, as compared to the control (T0). With the exception of available P, inorganic fertilizer (T1) did not significantly affect soil chemical parameters as compared to the control (T0).

**Table 4:** Correlation coefficient between yield parameters of cassava and soil properties.

	BD	TP	WHC	pH	OM	N	P	K	Ca	Mg
FTY	-0.718**	0.716**	0.741**	0.330**	0.746**	0.814**	0.567**	0.857**	0.790**	0.775**
ADB	-0.660**	0.658**	0.689**	0.328**	0.715**	0.719**	0.586**	0.843**	0.752**	0.743**

*Significant difference at p = 0.05.

**Significant difference at p = 0.01.

### *Titthonia diversifolia* fresh biomass and poultry manure effect on Yield parameters of cassava

**Figure 2:**
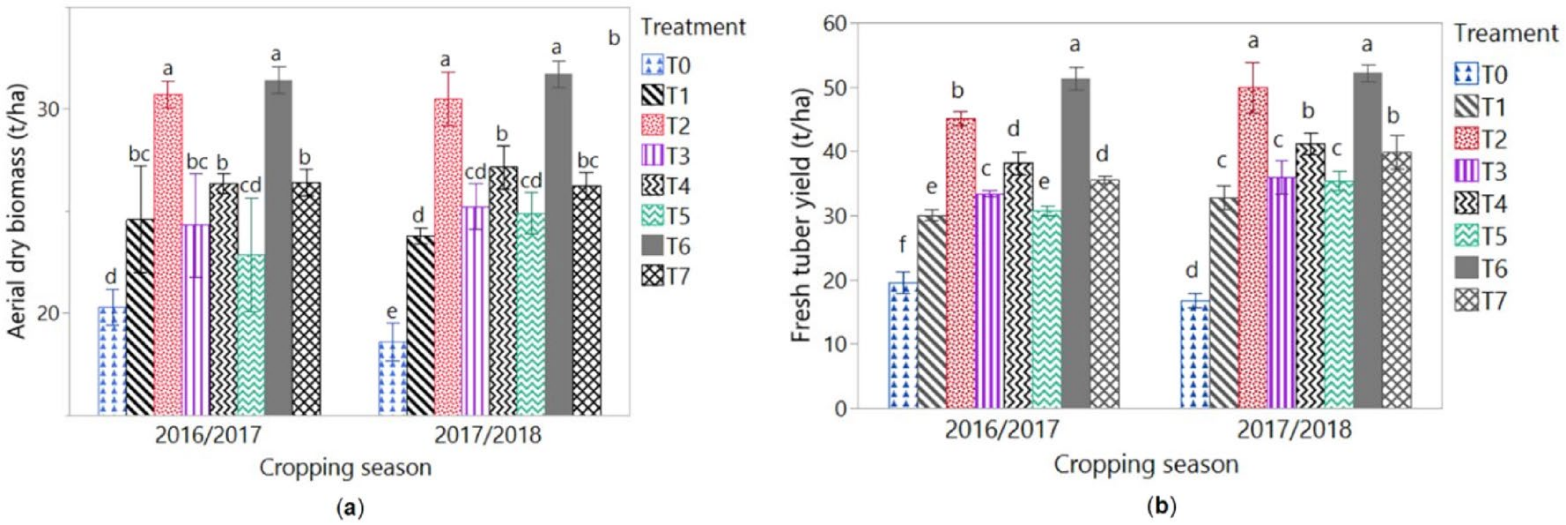
Means of aerial dry biomass (**a**) and fresh tuber yield (**b**) of cassava in 2016/2017 and 2017/2018 cropping seasons. T0 = control (no amendement), T1 = IF : 13-13-23 NPK at 450 t ha^−1^ + Urea at 100 t ha^−1^, T2 = TB at 20 t ha^−1^, T3 = TB at 10 t ha^−1^, T4 = PM at 20 t ha^−1^, T5 = PM at 10 t ha^−1^, T6 = TB at 10 t ha^−1^ + PM at 10 t ha^−1^, T7 = TB at 5 t ha^−1^ + PM at 5 t ha^−1^. *TM* *Tithonia diversifolia* fresh biomass, *PM* poultry manure, *IF* inorganic fertilizer. Values followed by similar letters are not significantly different at p = 0.05.

Yield parameters of cassava were significantly affected by the amendments applied (Fig. 2). Applied solely or mixed, *Tithonia diversifolia* fresh biomass and poultry manure improved ADB and FTY as compared to the control (T0). Similarly, treatments with inorganic fertilizer (T1) increased ADB and FTY of cassava as compared to the control (T0).

Over both cropping seasons, an increasing trend of the yield parameters was observed with increasing rates of organic manures. The highest yield occurred with the application of TB at 20 t ha^−1^ (T2), PM at 20 t ha^−1^ (T4) and TB + PM at 10 t ha^−1^ each (T6). Globally, ABD and FTY increased in the range from 22 to 60% and 77 to 172%, respectively, as compared to the control (T0) when *Tithonia diversifolia* fresh biomass and poultry manure, were applied solely or mixed at different rates (T2, T3, T4, T5, T6, and T7). Furthermore, ABD and FTY was increased by 23 and 69%, respectively, in the inorganic fertilizer treatment as compared to the control (Fig. 2). Significant changes were also noticed on aerial dry biomass and fresh tubers yield of cassava in the 2016/2017 and 2017/2018 cropping seasons. Cassava aerial dry biomass and fresh tubers yield performed better in 2017/2018 cropping season. Taken as individual factors, cropping season (S) was not significant for aerial dry biomass (ADB) and fresh tuber yield (FTY). Treatment (T) was significant for all yield parameters (ADB and FTY). The S × T interaction was not significant for cassava aerial dry biomass and cassava fresh tuber yield.

### Correlations between cassava yield parameters and selected soil properties

The study also revealed that cassava yield parameters depended on soil physical and chemical properties (Table 4). A negative and strong correlation was noticed between fresh tubers yield of cassava and bulk density, while total porosity and water holding capacity showed a positive and strong correlation with cassava fresh tubers yield. Likewise, a positive and strong correlation was also noted between yield parameters of cassava (aerial dry biomass and fresh tubers yield) and soil organic matter, total nitrogen and exchangeable cations (K, Ca and Mg).

### Soil quality index under different treatments in 2016/2017 and 2017/2017 cropping seasons.

**Figure 3:**
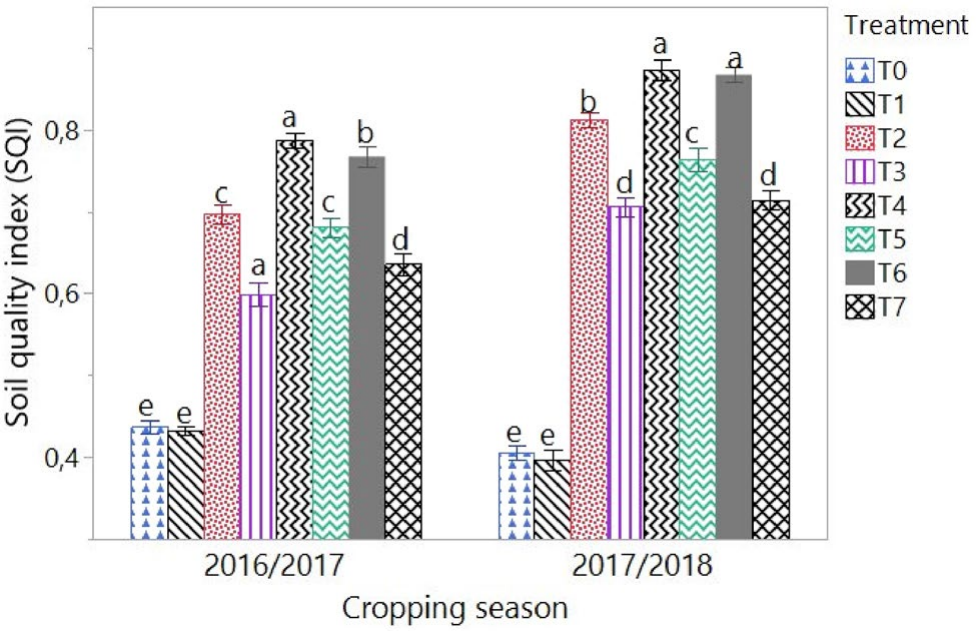
Variation in soil quality index (SQI) under different treatments in the 2016/2017 and 2017/2018 cropping seasons. T0 = control (no amendement), T1 = IF : 13-13-23 NPK at 450 t ha^−1^ + urea at 100 t ha^−1^, T2 = TB at 20 t ha^−1^, T3 = TB at 10 t ha^−1^, T4 = PM at 20 t ha^−1^, T5 = PM at 10 t ha^−1^, T6 = TB at 10 t ha^−1^ + PM at 10 t ha^−1^, T7 = TB at 5 t ha^−1^ + PM at 5 t ha^−1^. *TM* *Tithonia diversifolia* fresh biomass, *PM* poultry manure, *IF* inorganic fertilizer. Values followed by similar letters are not significantly different at p = 0.05.

With a total data set of 11 parameters (BD, TP, WHC, pH, SOM, N, P, K, Ca, Mg and CEC), N and SOM were retained in the minimal dataset and therefore represent the parameters that best explain soil fertility in the treatments. Figure 3 below shows that the soil quality index (SQI) increased with the cropping seasons in all plot treated with organic manures (T2 to T7), while it remained stable in the control (T0) and inorganic fertilizer (T1) treatments. In 2016/2017 cropping season, the SQI values varied from 0.43 to 0.79 with an average of 0.63, and from 0.34 to 0.87 with an average of 0 0.69 in 2017/2018 cropping season. T4 and T6 had the highest SQI values regardless of the cropping season with SQI values of 0.79 and 0.77 for the 2016/2017 cropping season and 0.87 and 0.86 for the 2017/2018 cropping season respectively. In general, the soil quality index has a concave evolution from T0 to T7 during the two cropping seasons.

**Figure 4:**
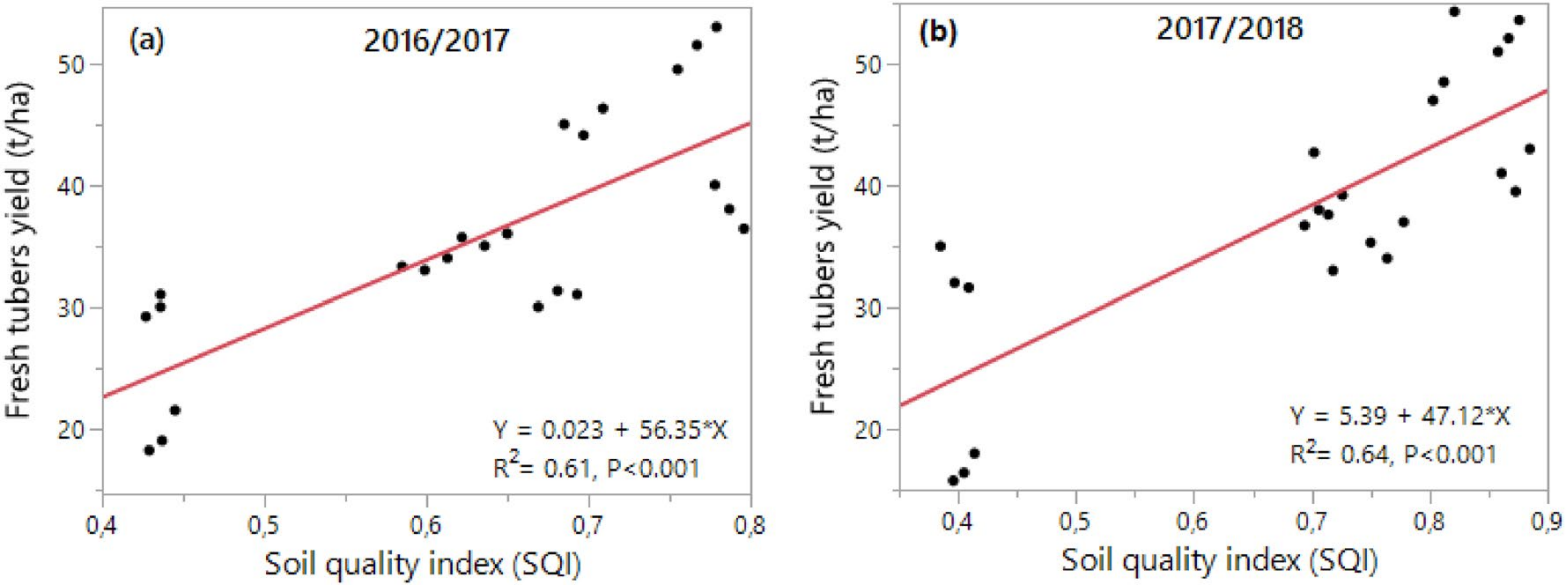
Relationships between Soil Quality Index (SQI) values and cassava yield in the 2016/2017 and 2017/2018 cropping seasons.

In addition, the correlation between SQI and cassava fresh tubers yield was significant (P < 0.05) in the two-studied cropping seasons 2016/2017 and 2017/2018 (Fig. 4).

### Economic return of the application of the application of *Titthonia diversifolia* fresh biomass and poultry manure

The economic analysis data for the production of cassava under the different treatments recorded in Table 5 showed that the application of 20 t ha^−1^ of poultry manure (T4) accounted for 33.3% of the total cost of production followed by 26.5% of *Titthonia* biomass mixed with poultry manure applied at 10 t ha^−1^ each (T6) while the least was 13.3% for *Titthonia* biomass applied at 10 t ha^−1^. With regards to the economic performance, the result obtained from *Titthonia* biomass mixed with poultry manure applied at 10 t ha^−1^ each (T6) indicated a maximum gross returns of FCFA 5 436 900.00 ha^−1^, net returns of FCFA 3 736 900.00 ha^−1^ and benefit:cost ratio of 3.2:1. The highest profit of FCFA 2.3 per franc CFA invested among the various treatments was recorded with the application of 20 t ha^−1^ of *Titthonia* biomass. The control treatment (T0) gave the least gross return of FCFA 1 904 700.00 ha^−1^ and net returns of FCFA1 099 700.00 ha^−1^ while the least benefit:cost ratio and profit were recorded with poultry manure applied at 20 t ha^−1^ (T4) with 2.3:1 and FCFA1.3 per franc CFA invested.

**Table 5:** Economics of producing cassava under each treatment for cropping seasons 2016/2017–2017/2018.

Treatment code	Gross return (FCFA ha^−1^)	Fertilizer cost (FCFA ha^−1^)	Total cost of production (FCFA ha^−1^)	Net return (FCFA ha^−1^)	Benefit/cost ratio	Returns/FCFA outlay
T0	1,904,700	–	800,500	1,099,700	2.4:1	1.4
T1	3,304,350	265,500	1,240,500	2,063,850	2.7:1	1.7
T2	4,990,650	300,000	1,525,000	3,465,650	3.3:1	2.3
T3	4,163,250	600,000	1,800,000	2,363,250	2.3:1	1.3
T4	5,436,900	450,000	1,700,000	3,736,900	3.2:1	2.2
T5	3,639,300	150,000	1,125,000	2,514,300	3.2:1	2.2
T6	3,475,500	300,000	1,300,000	2,175,500	2.7:1	1.7
T7	3,958,500	225,000	1,350,000	2,608,500	2.9:1	1.9

Notes: T0 = control (no amendement), T1 = IF : 13-13-23 NPK at 450 t ha^−1^ + urea at 100 t ha^−1^, T2 = TB at 20 t ha^−1^, T3 = TB at 10 t ha^−1^, T4 = PM at 20 t ha^−1^, T5 = PM at 10 t ha^−1^, T6 = TB at 10 t ha^−1^ + PM at 10 t ha^−1^, T7 = TB at 5 t ha^−1^ + PM at 5 t ha^−1^.

*TM* *Tithonia diversifolia* fresh biomass, *PM* poultry manure, *IF*  inorganic fertilizer In 2017, the price of cassava fresh tuber was FCFA35·kg^−1^. In 2018, the price of cassava fresh tuber was FCFA70·kg^−1^. Urea (46% N) was FCFA320·kg^−1^ ; NPK (13, 13, 23) was FCFA518·kg^−1^. FCFA612 = US$1.00 in 2017; FCFA534 = US$1.00 in 2018.

## Discussion

The results of this study showed a reduction in soil bulk density and an increased in water holding capacity and porosity in sole *Tithonia diversifolia* fresh biomass (TB) and poultry manure (PM) or their combined treatments as compared to the inorganic fertilizer (IF) and the control treatments. The amelioration of these soil physical properties by both organic manures (TB and PM) was probably due to increase in soil organic matter^20–22^. The observed trend of the decrease in soil bulk density^23–25^ and increase in soil total porosity^15,26,27^ by raising the proportions of organic manure have been reported respectively. Sharma et al.^28^, found an enhancement in soil physical properties such as water retention and aggregate stability under subtropical conditions due to addition of organic manures. Application of inorganic fertilizer has not shown any evidence of soil physical parameters improvement because of its lack of organic matter, as reported earlier by Hafifah et al.^20^ and Kolawole et al.^23^.

The results showed that TB and PM, applied solely or mixed, improved soil chemical properties as compared to the control. This showed that the application of organic manures released nutrients into the soil following their degradation by soil biota. Agbede et al.^21^ and Kolawole et al.^23^ noted significant improvement of soil chemical properties such as pH, N, OM, P, K, Mg and CEC on application of organic manures such as *Tithonia diversifolia* green manure and poultry manure in West African soils. During the experiment, soil pH increased with the application of TB and PM, but decreased with inorganic fertilizer. The reduction of pH in plots amended with inorganic fertilizer would certainly be due to the acidifying nature of the fertilizers used^29^. The ability of organic manures to increase soil pH was studied by Duruigbo et al.^30^ who related it to the presence of base cations contained in these organic manures.

Soil nutrient levels were higher in plots amended with TB + PM, which might be as a result of the good chemical composition of these two organic manures. Indeed, given the low C/N ratio of TB and the relatively high C/N ratio of PM, the application of TM combined with PM has induced good mineralization and better release of nutrients compared to their single forms. These results comply with those of Biratu et al.^17^ and Adekiya et al.^31^ who reported that the quality of organic matter is related to its chemical composition and C/N ratio, and the mixture of low and high C/N organic residues impacts soil fertility better.

The improvement of cassava yield with *Tithonia diversifolia* fresh biomass (TB) and poultry manure (PM) applied solely or combined was as a result of improved soil physical and chemical parameters. The poor performance of cassava recorded from the unamended plots (control) was probably due to their low content in soil organic matter and nutrients. Plots treated with organic manures (TB and PM or both mixed) performed better than those treated with inorganic fertilizer (IF) because of the suitable soil physical conditions created by the soil organic matter and the nutrients release from organic fertilizers^21,32^.

Apart from soil physical and chemical properties, organic manures also increased cassava yield parameters (aerial dry biomass (ADB) and fresh tuber yield (FTY)). The high levels of the yield parameters of cassava were recorded on plots treated with TB and PM in the ensuing cropping season of 2017/2018. This was probably due to the accumulation effects after repeated addition of organic fertilizers. These findings were confirmed by those of Biratu et al.^17^ and Kolawole et al.^23^ who noticed higher yield parameters of cassava with increasing levels of different organic amendments. The best performance of cassava was recorded with TB + PM applied at 10 t ha^-1^ each which might be related to better soil physical and chemical conditions created by this mixture of organic fertilizers. Our findings agreed with those of Agbede et al.^32^, who observed significant improvement of yam yield due to the application of a mixture of green manure and poultry manure in Nigeria. Also, Al-Gaadi et al.^33^ in the central region of Saudi Arabia and Biratu et al.^17^, reported that crop yields were better with the application of organic fertilizers such as poultry manure.

The strong correlation observed between cassava yield parameters and soil physical (BD, TP and WHC) and chemical properties (OM, N, K, Ca and Mg) indicated that the yield of cassava was strongly depend on these soil properties. Bakayoko et al.^34^ and Agbede^35^ earlier reported that the performance of crops such as cassava was strongly affected by soil physical and chemical parameters which might influence root penetration and nutrient uptake.

According to the classification of Li et al.^36^, soil quality index (SQI) was high on plots treated with organic manure and low on control and inorganic fertilizers. The SQI values increased with application of organic manure. Increasing rates of organic manure application led to an increasing trend in SQI values, and significant differences were noticed among the various manure application rates. The increase in SQI values seems to be in agreement with the general increase in crop yield with increasing rates of organic manure application. Cen et al.^37^ and Shashid et al.^38^ have demonstrated that the application of manure leads to an upward trend in SQI values. The study revealed a significant correlation between SQI values and cassava yield (Fig. 4). A positive correlation (R2 = 0.61 in 2016/2017 and R2 = 0.64 in 2017/2018) between index values and yield implied that the index may have practical utility in quantifying the soil quality under cassava cultivation.

The differences in the variable costs observed are attributable to the type of fertilizers and different proportions applied. Poultry manure applied at 20 t ha^−1^ was not very beneficial, and cassava production was expensive with this treatment. Average of 51.78 t ha^−1^ of cassava fresh tubers was obtained with the mixture of Tithonia and poultry manure applied at 10 t ha^−1^ each, it was high enough to compensate the cost of the production investment and also the higher net return of FCFA 3 736,900.00 ha^−1^ related with it. These findings were confirmed by those of Agbede et al.^32^ who recorded very high yield of yam with a mixture organic fertilizers applied at optimum to compensate the high cost of production invested.

The benefit:cost ratio of 3.2:1 obtained from the application of the mixture of *Tithonia diversifolia* fresh biomas and poultry manure both at 10 t ha^−1^ each, indicated that this treatment was the most profitable for cassava production. It can be shown that, all the amended treatments were profitable but TB + PM at 10 t ha^−1^ each was the most profitable and sustainable.

## Materials and methods

### Study sites

The study was carried out at Essong-mintsang (04°05′02″N Latitude and 11°35′09″E Longitude) in southern Cameroon, during two cropping seasons: The first (2016/2017) between march 2016 and march 2017, and the second (2017/2018) between march 2017 and march 2018. The annual rainfall is between 1300 and 2000 mm and the mean temperature is between 23 and 26 °C. The area is characterized by a bimodal rainfall pattern, with four seasons, such that the major and minor rainy seasons last from mid-March to early July and from mid-August to mid-November, respectively^39^. The experimental field had a gentle slope covered with a young fallow dominated by *Chromolaena odorata*. The soil types are dominated by Ferralsols according to WRB^19^, with the risk of seasonal flooding in the depth as a result of their hydromorphic nature^40^. The locality Essong-Mintsang lies in the forest savannah transitional zone of the rainforest region and now has a more or less semi-deciduous type of vegetation instead of primary evergreen forest.

### Plant material

All experiments and field studies on plants complied with relevant institutional, national, and international guidelines and legislation. The plant material used in this study consisted of an improved cassava variety, namely 8034 obtained from the Institute of Agricultural Research for Development. It is renowned for its resistance to African cassava mosaic disease and bacterial blight and its tolerance to mealy bugs and bacterial blight. The average yield of the variety 8034 is between 30 and 40 t ha^−1^ of fresh roots with a dry matter content of approximately 35–38%^41^.

### Experimental design

A randomized complete block design (RCBD) replicated in three blocks with 8 treatments was conducted. *Tithonia diversifolia* fresh biomass (TB) and poultry manure (PM) were used as organic manures while NPK (13 13 23) purchased from the local market (Mfoundi-Yaounde, Cameroon) was used as the inorganic fertilizer (IF). The treatment details are given in Table 6.

**Table 6:** Experimental treatments with organic manures and inorganic fertilzer.

No	Treatment code	Treatment type	Treatment rates
1	T0 (control)	–	–
2	T1	IF (13-13-23 NPK + Urea)	450 kg ha^−1^ + 100 kg ha^−1^
3	T2	TB	20 t ha^−1^
4	T3	TB	10 t ha^−1^
5	T4	PM	20 t ha^−1^
6	T5	PM	10 t ha^−1^
7	T6	TB + PM	10 + 10 t ha^−1^
8	T7	TB + PM	5 + 5 t ha^−1^

*TB* *Tithonia diversifolia* fresh biomass, *PM* poultry manure, *IF* inorganic fertilizer.

*Tithonia diversifolia* fresh biomass and poultry manure were chosen as soil amendment because of their high nutrients content^18,22,42^. The IF (450 kg ha^−1^ 13-13-23 NPK + 100 kg ha^−1^ Urea) used was equivalent to 100 N:22P:83 K kg ha^−1^ which is the inorganic fertilizer rate recommended by Howeler, et al.^14^ for cassava production. Poultry manure was collected from poultry farms around the study area. *Tithonia diversifolia* fresh biomass (fresh leaf with petiole and soft stem) was collected locally.

### Sowing and amendment

Cassava was planted at the rate of one cutting of 25–30 cm in length per hole following an intra-row spacing of 1 m and inter-row spacing of 1 m. The cuttings were planted in such a way that 2/3 of the cutting was below ground and 1/3 above ground level with a 45° inclination. The organic manures (*Tithonia diversifolia* fresh biomass and poultry manure) were dropped into the soil at 20 cm depth with a hoe two weeks before planting. The inorganic fertilizers were applied in two replications: 50 kg ha^−1^ at planting and 50 kg ha^−1^ at 1 months after planting (MAP) as urea. While 13-13-23 NPK was at the rates of 225 kg ha^−1^ at 1 MAP and the remaining 225 kg ha^−1^ at 3 MAP. Manual weeding was done as required. Harvesting was done 12 months after planting. Water during the entire experimental period depended on the local rainfall regime of the study site.

### Sampling and analysis

Prior to incorporation of organic fertilizers to plots, samples were taken for chemical analysis (C, N, P, K, Ca and Mg) as described by Tel and Hagarty^43^.

Composite samples of the top soil (0–20 cm) were collected from the experimental field with an auger before seed bed preparation. About 300 g of the samples were taken for physical and chemical anlysis in the laboratory. Soil particle size analysis was done by pipette method^44^. Soil bulk density (BD) was determined by the core method^45^. Soil water holding capacity (WHC) was determined following the method of ISO 11274^46^. Soil pH (1:5 solution) was determined in a 1:5 (w/v) soil to water solution using a pH meter as outlined by McLean^47^. Soil organic carbon (OC) was determined by Walkley and Black method^48^. Organic matter (OM) was calculated by multiplying OC by 1.724. Total N was determined using the Kjeldahl method^49^ and available phosphorus (P) was determined by Bray-2 extraction method^50^. CEC was extracted using ammonium acetate method^51^. Exchangeable K, Ca and Mg were extracted with a 1 M NH_4_OAc, pH 7 solution. Thereafter, K was analyzed with a flame photometer and Ca and Mg were determined with an atomic absorption spectrophotometer.

### Determination of yield parameters of cassava

The yield data of cassava were obtained from randomly selected six plants per plot. The aerial dry biomass (ADB) and fresh tuber yield (FTY) were measured as yield parameters at harvest (12 MAP).

### Soil quality index

The determination of the soil quality index (SQI) was done in three steps^52,53^ namely (1) the selection of the minimum data set through the principal component analyzes and Pearson correlations for the choice of parameters that best characterize soil fertility, (2) the assignment of scores ranging from 0 to 1 to each parameter of the minimum data set and (3) the combination of scores into an index according to the following equation:

$$ {\text{SQI}} = \sum\limits_{{{\text{i}} = 1}}^{{\text{n}}} {{\text{Wi}} \times {\text{Si}}} $$

where, *Wi* is the assigned weight of each indicator, *Si* is the indicator score, and *n* is the number of variables.

### Statistical analyses

Data collected from each experiment were subjected to analysis of variance (ANOVA) and treatment means were compared using the Duncan’s multiple range test at P ≤ 0.05. Pearson’s correlation was used to investigate the correlation between Cassava yield parameters and soil properties. All statistical analysis were performed using the Statistical Package for the Social Sciences (SPSS) version 17.0 (Chicago: SPSS Inc., 2008).

### Economic analysis

The economic analysis was done in order to determine the net farm income, benefit/cost ratio and outlay for cassava production. Cost benefit analyses were determined in franc CFA (1$US = FCFA 612.00 Cameroonian currency in the year 2017 and FCFA 534 in the year 2018). The net farm return (Nfr) is the differences between total revenue (Tr) obtained based on the average market retail prices for the period considered, and total cost of production (Tcp). Average market price were recorded from Mfoundi, Etoudi and Nkol-Eton local markets in Yaounde, Cameroun. All the calculations were performed using the following formula :

$$ \begin{gathered} {\text{Net farm return }}\left( {{\text{Nfr}}} \right) \, = {\text{ Tr }}{-}{\text{ Tcp}} \hfill \\ {\text{Total revenue }}\left( {{\text{Tr}}} \right) \, = {\text{ P }} \times {\text{ Q}},{\text{ where P is the prevailing market price and Q is the quantity of produce or fresh tubers yield}} \hfill \\ {\text{Benefit}}/{\text{cost ratio }} = {\text{ Tr}}/{\text{Tcp}} \hfill \\ {\text{Return per franc CFA invested }} = {\text{ Nfr}}/{\text{Tcp}} \hfill \\ \end{gathered} $$

### Ethics approval and consent to participate

This manuscript is an original paper and has not been published in other journals. The authors agreed to keep the copyright rule.

### Consent for publication

The authors agreed to the publication of the manuscript in *Scientic Reports*.

## Conclusion

The study showed that application of organic manures improved soil physical and chemical properties by lowering soil bulk density, increasing total porosity, water holding capacity, soil pH, organic matter, available P, total N, CEC and exchangeable nutrients (K, Ca and Mg), and exhibited high SQI. This indicated an improvement in soil fertility and quality which resulted in a significant increase in yield. SQI was positively and significantly correlated with cassava yield under all the two-studied cropping seasons. This means that the index parameters are useful for computing the soil quality under cassava cultivation. Application of the mixture of *Tithonia diversifolia* fresh biomass and poultry manure at 10 t ha^-1^ each (T6), was the optimum rate of producing cassava, which would support the demand placed on the soil to produce higher yield without deleterious effect on the soil and the environment. Likewise, it is the most profitable and cost-effective rate of producing cassava that would be beneficial to smallholder farmers in the humid forest zone of southern Cameroon.

## Data Availability

The datasets generated during and/or analysed during the current study are available from the corresponding author on reasonable request.
